# Insulin‐Like Growth Factor 2 mRNA‐Binding Protein 2 (IGF2BP2) Promotes Castration‐Resistant Prostate Cancer Progression by Regulating AR‐V7 mRNA Stability

**DOI:** 10.1002/cnr2.70096

**Published:** 2025-02-13

**Authors:** Taruna Saini, Devesh Srivastava, Rajnikant Raut, Parul Mishra, Ashish Misra

**Affiliations:** ^1^ Department of Biotechnology Indian Institute of Technology Hyderabad Kandi Sangareddy India; ^2^ Department of Animal Biology School of Life Sciences, University of Hyderabad Hyderabad India

**Keywords:** androgen receptor, AR‐V7, castration‐resistant prostate cancer, IGF2BP2, RNA‐binding proteins

## Abstract

**Background:**

The emergence of constitutively active androgen receptor (AR) splice variant AR‐V7 poses a formidable challenge in treating prostate cancer, as it lacks the ligand binding region targeted by androgen‐deprivation therapies such as enzalutamide and abiraterone. AR‐V7 is critical for castration‐resistant prostate cancer (CRPC) development and progression; however, the molecular mechanisms regulating its expression and biological function remain poorly understood. Here, we investigate the role of IGF2BP2 in regulating AR‐V7 expression and CRPC progression.

**Methods:**

To determine the clinical relevance of IGF2BP2 in CRPC, we analyzed the mRNA expression data for prostate cancer patients available in the Genomic Data Commons (GDC) Data Portal and cBioPortal. Next to investigate the role of IGF2BP2 in regulating AR‐V7 expression and enzalutamide resistance, we performed shRNA‐mediated IGF2BP2 knockdown and overexpression experiments followed by qRT‐PCR, immunoblot, colony‐formation, and MTT assays. Finally, we performed RIP‐qPCR, actinomycin‐D, and IGF2BP2 domain‐deletion analysis to study the mechanism by which IGF2BP2 regulates AR‐V7 stability, expression, and enzalutamide resistance in CRPC cells.

**Results:**

Our analysis revealed that IGF2BP2 is upregulated in CRPC patients and its expression positively correlates with increasing Gleason score in patients with CRPC. We demonstrate that IGF2BP2 silencing leads to downregulation of AR‐V7 and its downstream target genes without affecting AR levels. Additionally, IGF2BP2 knockdown also enhances the sensitivity of CRPC cells to enzalutamide while overexpression increases AR‐V7 expression and confers increased resistance to enzalutamide. Mechanistically, our experiments demonstrate that IGF2BP2 binds to the intronic splicing enhancer (ISE) region of AR‐V7, thereby enhancing its mRNA stability. Furthermore, our domain‐deletion analysis pinpoints the role of KH3 and KH4 domains of IGF2BP2 in regulating AR‐V7 stability and enzalutamide resistance.

**Conclusions:**

Taken together, our findings suggest that IGF2BP2 plays a critical role in regulating AR‐V7 expression and stability, offering a novel target for developing therapeutic interventions for CRPC.

## 1 Introduction

Prostate cancer is the second deadliest cancer among men worldwide. The growth and survival of prostate cancer depends on the steroid nuclear receptor termed androgen receptor (AR). It belongs to the hormone receptor family, and comprises of 8 exons that encode for N‐terminal, DNA binding, hinge region, and the ligand binding domains [1]. Androgen‐deprivation therapy (ADT), employing medications such as abiraterone and enzalutamide [2], serves as an AR‐targeted approach to impede the advancement of prostate cancer. Abiraterone impairs AR signaling by modulating androgen synthesis, while enzalutamide blocks AR activity by binding to LBD. Although initially effective, patients quickly develop resistance to these therapies, leading to the emergence of CRPC [3]. While multiple pathways are known to contribute to CRPC progression, the emergence of androgen receptor splice variant, AR‐V7, is believed to be the key driver of CRPC [4, 5, 6, 7]. Clinical studies have unequivocally demonstrated that increased AR‐V7 expression relates to poor prognosis in CRPC patients [8, 9].

Numerous post‐transcriptional regulators have emerged as pivotal players in regulating AR‐V7 generation and activity in CRPC, offering promising avenues for therapeutic intervention. Among these regulators, RNA‐binding proteins (RBPs) are known to play a critical role in modulating mRNA processing, polyadenylation, stability, and translation [10, 11]. Extensive studies have underscored the importance of RBPs in CRPC development and progression [12, 13]. The expression of heterogeneous nuclear ribonucleoprotein A1 (hnRNPA1) and Sam68 positively correlates with AR‐V7 generation and expression in prostate cancer [14, 15]. In fact, the molecular circuitry involving hnRNPA1, NF‐κB2/p52, and c‐Myc, intricately modulates AR‐V7 generation in prostate cancer cells [14]. hnRNPH1 and hnRNPL have also been shown to affect AR splicing and contribute to CRPC development [16, 17]. PTB‐associated splicing factor (PSF) has been shown to promote generation of AR and its splice variants by modulating the spliceosome machinery [18]. Therefore, the discovery of previously undocumented RBPs driving AR‐V7 expression will provide alternative treatment options for CRPC.

N6‐methyladenosine (m6A) is a dynamic internal modification within eukaryotic mRNAs, influencing gene expression by modulating RNA stability, subcellular localization, translation efficacy, and alternative splicing [19]. This modification has emerged as a critical regulator of CRPC progression and enzalutamide resistance. m6A levels are elevated in CRPC specimens compared to those of castration‐sensitive prostate cancer and altered m6A levels are known to affect the expression and splicing of AR‐V7 inside cells [20, 21]. Methyltransferase‐like 3 (METTL3), an integral component of the m6A “writer” methyltransferase complex, is upregulated in PCa, and its increased expression promotes tumor growth, correlating with advanced tumor staging and poor prognosis [22, 23]. It also contributes to CRPC progression and enzalutamide resistance by regulating m6A modification of genes involved in the ERK pathway [21]. Selective recruitment of m6A writer proteins to m6A sites on RNA is critical for ensuring the effect of m6A modification inside cells. IGF2BP2, a m6A reader protein, is a member of the IGF2 mRNA‐binding protein family that regulates various post‐transcriptional processes such as mRNA localization, stability, and translation by recognizing the m6A modifications on transcripts critical for cancer initiation and progression [24, 25]. Structurally, IGF2BP2 is a 66 kDa protein comprising two‐RRMs domains at the N‐terminal and four‐hnRNP‐K homology (KH) domains at the C‐terminus [26]. Studies have shown that IGF2BP2 dysregulation is associated with various diseases, including cancer, diabetes, and insulin resistance [26]. Overexpression of IGF2BP2 is known to promote tumor progression in a variety of malignancies, such as glioblastoma and colorectal cancer [27, 28, 29]. Recently, it has been reported that VIM‐AS1/IGF2BP2/HMGCS1 axis plays a critical role in regulating CRPC progression and enzalutamide resistance [30].

Here, we demonstrate that IGF2BP2 is significantly upregulated in CRPC patients and promotes CRPC cell proliferation by regulating AR‐V7 expression. Knockdown of IGF2BP2 results in downregulation of AR‐V7 and its downstream target genes. Furthermore, our experiments highlight the involvement of IGF2BP2 in proliferation of CRPC cells, stemness properties as well as tumorigenesis and establish a link between IGF2BP2 expression and enzalutamide resistance in CRPC cells. Mechanistically, we show that IGF2BP2 binds directly to ISE region of AR‐V7 pre‐mRNA via its KH3 and KH4 domains and plays a critical role in maintaining AR‐V7 mRNA stability.

## 2 Results

### 2.1 IGF2BP2 Regulates AR‐V7 Expression

**FIGURE 1:**
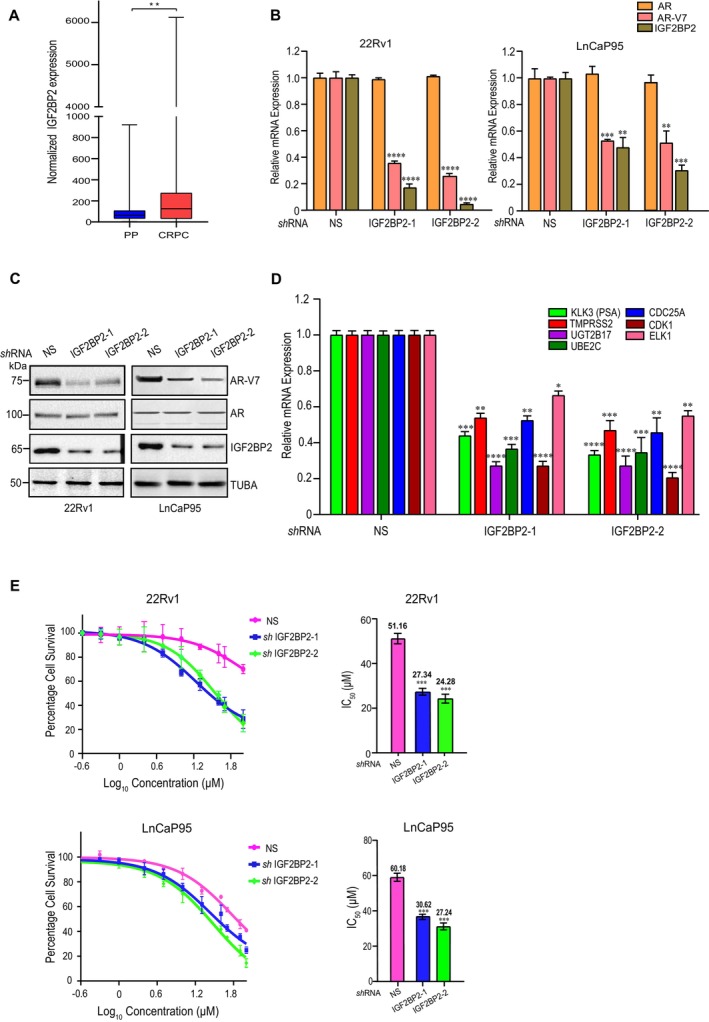
IGF2BP2 regulates AR‐V7 expression. A. IGF2BP2 mRNA expression in primary prostate (PP) (*n* = 500) compared to CRPC (*n* = 99) patient samples from TCGA dataset (*p* < 0.01, logFC = 1.88). mRNA expression is graphically represented as box and whiskers plot with outliers. B. qRT‐PCR analysis of AR‐V7, AR, and IGF2BP2 mRNA expression in 22Rv1 and LnCaP95 cell lines expressing NS, IGF2BP2–1, and IGF2BP2–2 shRNAs. C. Immunoblot analysis of AR‐V7 and AR in 22Rv1 and LnCaP95 cell lines expressing NS, IGF2BP2–1, and IGF2BP2–2 shRNAs. Tubulin was used as the loading control. D. qRT‐PCR analysis of indicated genes upon NS or IGF2BP2 knockdown in 22Rv1 cells. E. MTT assay to determine IC50 of enzalutamide in 22Rv1 and LnCaP95 cell lines expressing NS, IGF2BP2–1 and IGF2BP2–2 shRNAs. The data are shown as the mean ± SD; *n* = 3 independent experiments, two‐tailed student's t‐test, *p < 0.05, **p < 0.01, ***p < 0.001.

To determine the clinical relevance of IGF2BP2 in CRPC, we analyzed the mRNA expression data for prostate cancer patients available in the GDC Data Portal (https://portal.gdc.cancer.gov/). The data comprised of normal prostate (*N* = 52) and primary prostate cancer (*N* = 498) samples from TCGA‐PRAD and CRPC (*N* = 99) samples from WCDT‐MCRPC. Our analysis revealed that IGF2BP2 is significantly upregulated in CRPC patient samples compared to primary prostate cancer samples (*p* < 0.01, logFC = 1.88) (Figure 1A). We also observed that IGF2BP2 is upregulated in CRPC patient samples relative to the normal prostate samples (logFC = 0.7, p‐value = 0.013) (Figure S1). Next, we analyzed IGF2BP2 expression in 168 tumor samples from CRPC patients available in cBioPortal (PRAD_SU2C_2019 study), classified based on their Gleason scores. Our analysis revealed that IGF2BP2 mRNA expression is significantly higher in samples with elevated Gleason score [7, 9, 10] compared to normal prostate samples (Figure S2). These results point toward a positive correlation between IGF2BP2 expression and CRPC progression.

Next, to probe the role of IGF2BP2 in AR‐V7 expression, we analyzed the expression of AR‐V7 and AR in LnCaP95 and 22Rv1 cells infected with two sequence‐independent shRNAs against IGF2BP2 (Figure 1B). Cells expressing non‐silencing (NS) shRNA were used as control. We observed that downregulation of IGF2BP2 resulted in a significant decrease in AR‐V7 expression in both qRT‐PCR (Figure 1B) and immunoblot analysis (Figure 1B). Notably, full‐length AR levels remain unchanged, pointing toward the specificity of IGF2BP2 in regulating AR‐V7 levels (Figure 1A,B).

AR‐V7 is a constitutively active AR‐variant that regulates the canonical AR target genes, along with an unique set of genes such as UGT2B17, UBE2C, CDC25A, CDK1, and ELK1 [31, 32, 33]. We examined the impact of IGF2BP2 knockdown on these target genes using real‐time PCR (qRT‐PCR). Our results demonstrate that the expression of AR‐V7‐regulated genes, UGT2B17, UBE2C, CDC25A, CDK1, ELK1, and prostate‐specific antigen (PSA)/KLK3, is significantly decreased upon IGF2BP2 knockdown (Figure 1D) suggesting that alterations in IGF2BP2 expression directly affect the expression of AR‐V7 target genes.

Next, to examine the impact of IGF2BP2 on resistance to anti‐androgen inhibitors (enzalutamide and bicalutamide), we measured the IC50 of enzalutamide in IGF2BP2 knockdown 22Rv1 and LnCaP95 cells compared to non‐silencing (NS) shRNA infected cells using MTT assay. Knockdown of IGF2BP2 in 22Rv1 and LnCaP95 cells led to substantial decrease in IC50 of enzalutamide relative to NS knockdown cells. The IC50 for enzalutamide decreased from 51.16 μM in NS‐infected 22Rv1 cells to 27.34 μM and 24.28 μM in IGF2BP2 knockdown 22Rv1cells. In LnCaP95 cells, the IC50 decreased from 60.18 μM NS‐infected cells to 30.62 μM and 27.24 μM in IGF2BP2 knockdown cells (Figure 1E). The greater decrease in IC50 values of enzalutamide in cells expressing IGF2BP2–2 shRNA compared to IGF2BP2–1 shRNA is consistent with the knockdown efficiency of IGF2BP2 in those cell lines, with IGF2BP2–2 shRNA exhibiting higher knockdown efficiency than IGF2BP2–1 shRNA. We also observe similar reduction in bicalutamide IC50 in 22Rv1 cells expressing IGF2BP2 shRNAs compared to non‐silencing shRNA expressing cells (Figure S3). Collectively, our results demonstrate the role of IGF2BP2 in regulating AR‐V7 expression and resistance to next‐generation anti‐androgen inhibitors (enzalutamide and bicalutamide) in CRPC cells.

### 2.2 IGF2BP2 Recruitment to Specific Splice Sites on AR‐V7 Pre‐mRNA Is Required to Maintain Stability

**FIGURE 2:**
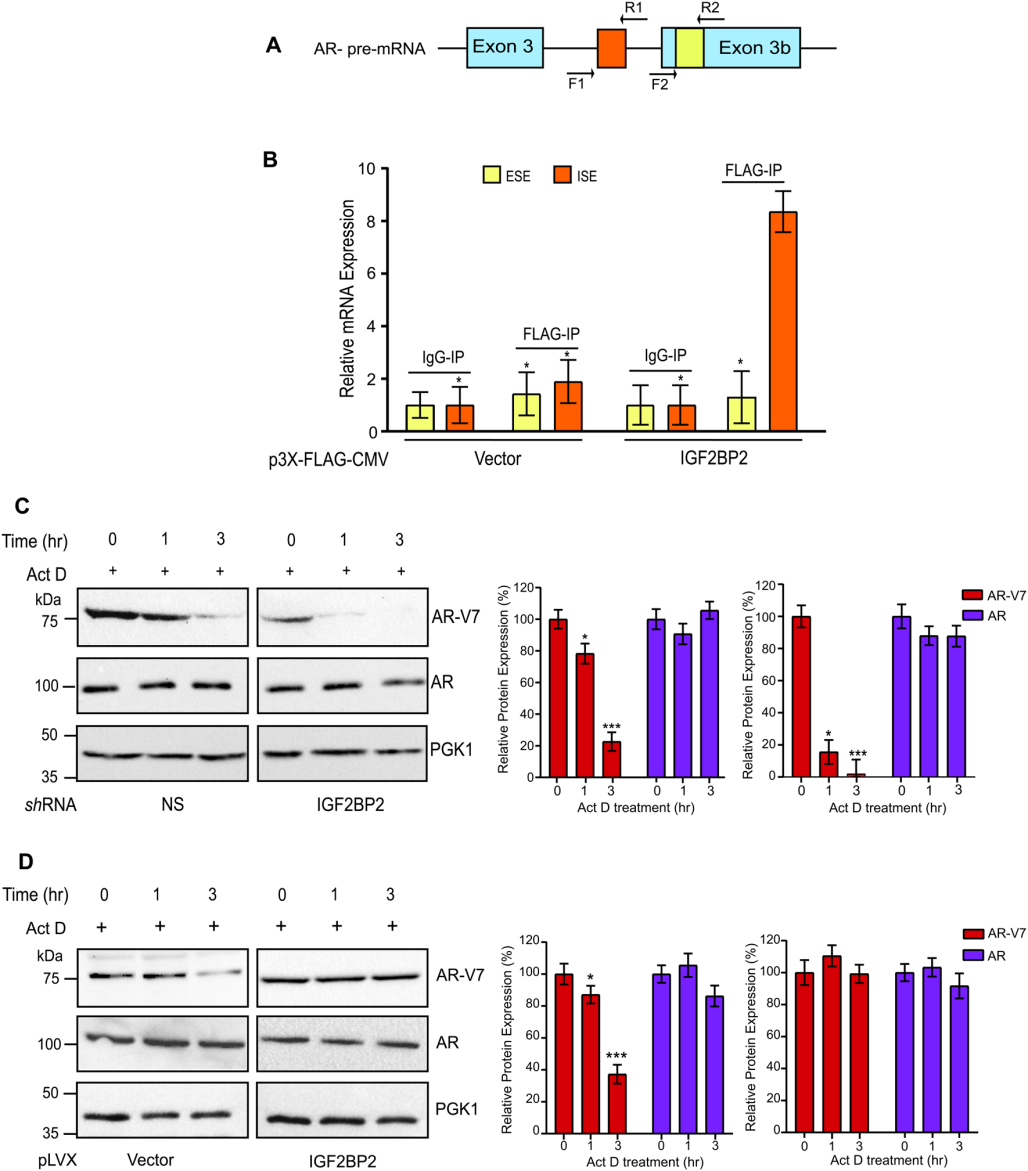
Recruitment of IGF2BP2 to specific splice sites on AR‐V7 pre‐mRNA is required to maintain stability A. Schematic representation of ISE and ESE sites on exon 3b of AR pre‐mRNA. F1‐R1 and F2‐R2 represent the qPCR primer locations in the ISE and ESE regions. B. RIP‐qPCR analysis showing enrichment of IGF2BP2 on ISE and ESE regions of AR‐V7 mRNA in IGF2BP2‐overexpressing VCaP cells relative to vector‐overexpressing cells. IgG was used as negative control. Fold‐enrichment relative to IgG for indicated sites is shown C. Immunoblot analysis of indicated proteins in 22Rv1 cells expressing IGF2BP2 or NS shRNAs following actinomycin‐D (5 μg/mL) treatment for 0, 1, and 3 h. (*n* = 3 biological replicates/group) D. Immunoblot analysis of indicated proteins in IGF2BP2‐overexpressing VCaP cells relative to vector‐overexpressing cells following actinomycin‐D (5 μg/mL) treatment for 0, 1, and 3 h. (*n* = 3 biological replicates/group). Densitometric analysis for relative AR‐V7 and AR expression was performed using Image J software and normalized to PGK1 expression. Data are the mean ± SD. *p < 0.05, **p < 0.01, ***p < 0.001.

Under androgen‐deprivation conditions, distinct splice sites on AR mRNA associate with various splicing factors and RBPs to regulate alternative splicing. Notably, intronic splicing enhancer (ISE) and exonic splicing enhancer (ESE) site near the 3′ splice site of exon 3b play a pivotal in AR‐V7 generation [34, 35] (Figure 2A). To determine, if IGF2BP2 is recruited to these splice sites, we performed an RNA immunoprecipitation assay followed by qRT‐PCR (RIP‐qPCR) using anti‐FLAG antibody in both FLAG‐IGF2BP2 and vector‐expressing VCaP cells. The RIP‐qPCR assay results revealed that IGF2BP2 is highly enriched in the ISE region compared to ESE region of AR‐V7 pre‐mRNA (Figures 2B and S4).

Next, to understand the effect of IGF2BP2 binding to AR‐V7 mRNA, we investigated the role of IGF2BP2 in regulating AR‐V7 stability. We assessed the half‐life of AR‐V7 protein upon actinomycin‐D treatment in 22Rv1 cells expressing either NS or IGF2BP2 shRNAs. Our immunoblot results revealed that AR‐V7 levels are significantly reduced in cells expressing IGF2BP2 shRNA relative to NS‐expressing cells 3 h post actinomycin‐D treatment, while no effect is observed on AR levels (Figures 2C and S5). This is consistent with enhanced AR‐V7 stability in VCaP cells expressing IGF2BP2 relative to vector‐expressing cells after actinomycin‐D treatment (Figure 2D). Collectively, our results demonstrate that IGF2BP2 directly binds to the ISE region of AR‐V7 mRNA and plays a crucial role in enhancing its stability.

### 2.3 IGF2BP2 Promotes Migration and Stemness in CRPC Cells

**FIGURE 3:**
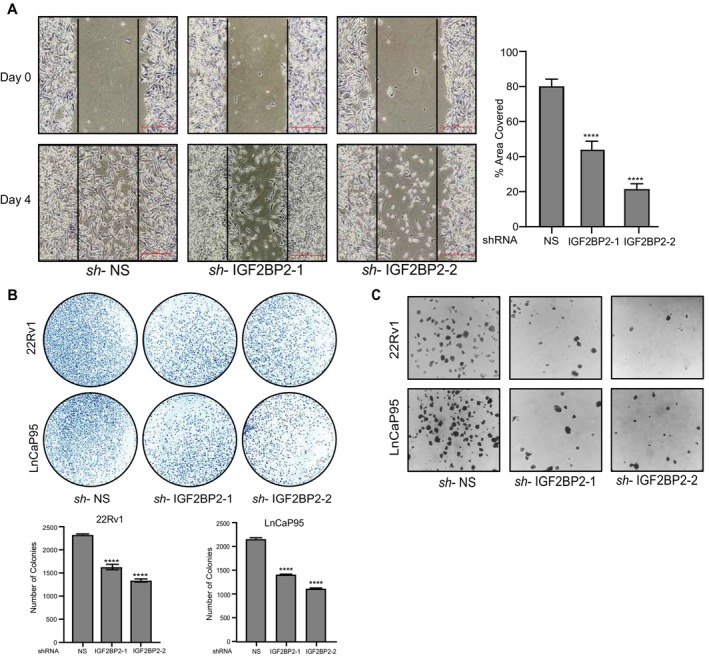
IGF2BP2 promotes migration and metastasis in CRPC cells. A. Wound‐healing assay in 22Rv1 cells expressing IGF2BP2 or NS shRNAs. Quantification of the total area covered by the cells was calculated in ImageJ software and plotted (****p < 0.0001). Scale bar, 100 μm. B. Colony‐formation assay in 22Rv1 and LnCaP95 cells expressing IGF2BP2 or NS shRNAs. Colonies were stained using crystal violet and counted (*n* = 3 biological replicates/group). Error bars indicate standard deviation and ****p < 0.0001 C. Representative stained images showing soft agar colony formation in 22Rv1 and LnCaP95 cell lines expressing IGF2BP2 or NS shRNAs. Images were taken after 21 days.

Cancer progression is marked by the ability of malignant cells to migrate and invade surrounding tissues [36]. We performed wound‐healing assay in NS and IGF2BP2 shRNA expressing 22Rv1 cells to assess the potential of IGF2BP2 in regulating migration properties of CRPC cells. Our results revealed a ~ 40–50 reduction in cell migration upon IGF2BP2 knockdown compared to NS (Figure 3A). Next, we assessed the effect of IGF2BP2 on colony‐formation ability of CRPC cells. Our results demonstrate that there is a substantial decrease in the number of colonies in 22Rv1 and LnCaP95 cell lines expressing IGF2BP2‐shRNAs compared to NS (Figure 3B). Moreover, reduced expression of IGF2BP2 is accompanied by the loss of expression of stemness‐ and EMT‐related genes such as Sox‐2, Oct‐4, NANOG, E‐cadherin, and Vimentin (Figure S6). These findings suggest a positive correlation between IGF2BP2 expression, stemness, and the colony‐formation ability of CRPC cells. Thereafter, we evaluated the ability of IGF2BP2 knockdown CRPC cell lines 22Rv1 and LnCaP to grow in an anchorage‐independent manner using soft agar colony‐formation assays. NS‐shRNA expressing CRPC cells were used as negative control. Our results demonstrate that IGF2BP2 knockdown leads to significant decrease in the ability of CRPC cells to form colonies in soft agar (Figure 3C).

Collectively, the above results highlight the involvement of IGF2BP2 in regulating CRPC cell migration, proliferation, colony formation, and anchorage‐independent growth, underscoring its role as a key player in CRPC progression.

### 2.4 IGF2BP2 Overexpression Enhances AR‐V7 Expression and Enzalutamide Resistance

**FIGURE 4:**
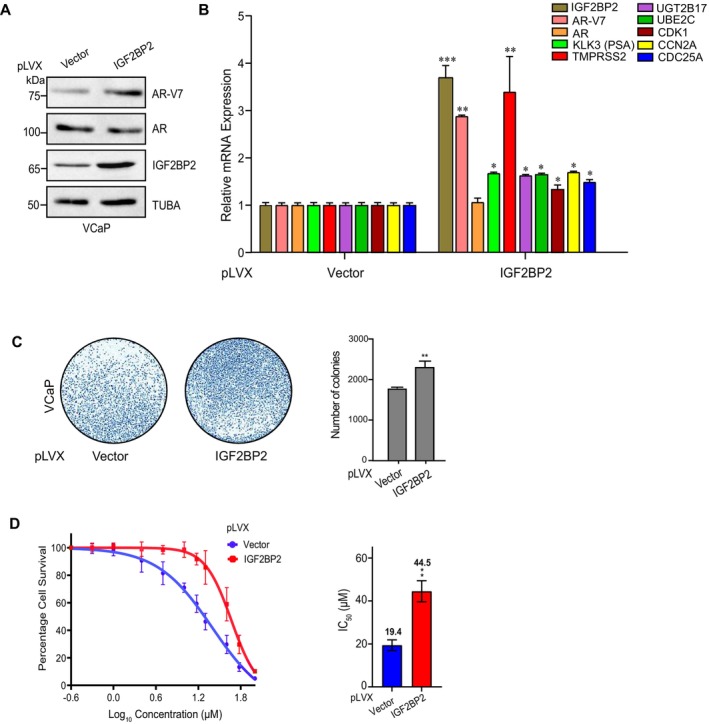
IGF2BP2 overexpression enhances AR‐V7 expression and enzalutamide resistance A. Immunoblot analysis of indicated proteins in IGF2BP2‐overexpressing VCaP cells relative to vector‐overexpressing cells. Tubulin was used as loading control. B. qRT‐PCR analysis showing mRNA expression of indicated genes in IGF2BP2‐overexpressing VCaP cells relative to vector‐overexpressing cells. C. Colony‐formation assay in IGF2BP2‐overexpressing VCaP cells relative to vector‐overexpressing cells. Colonies were stained using crystal violet, counted (*n* = 3 biological replicates/group) and mean ± SD (**p < 0.01) was plotted. D. MTT assay to determine IC50 of enzalutamide in IGF2BP2‐overexpressing VCaP cells relative to vector‐expressing cells. The data is shown as the mean ± SD; *n* = 3 independent experiments, two‐tailed Student's t‐test, **p < 0.01.

Next, we investigated the impact of IGF2BP2 overexpression on tumorigenic properties of VCaP cells. We infected VCaP cells with constructs overexpressing either empty vector or FLAG‐IGF2BP2 and monitored the levels of both AR and AR‐V7 using both qRT‐PCR and immunoblotting. Our results demonstrate that IGF2BP2 overexpression specifically enhances AR‐V7 levels inside cells while no effect is observed on full‐length AR levels (Figure 4A,B). Additionally, the expression of AR‐V7 downstream genes, including UGT2B17, UBE2C, CDC25A, CDK1, and ELK1, is markedly elevated in IGF2BP2‐overexpressing cells compared to vector‐expressing cells (Figure 4B). Consistent with these molecular changes, we also observe an increase in colony‐forming ability in cells overexpressing IGF2BP2 relative to vector‐expressing cells (Figure 4C). Next, we assessed the impact of IGF2BP2 overexpression on enzalutamide sensitivity of VCaP cells using MTT assay. Our results demonstrate that IGF2BP2‐overexpression enhances enzalutamide IC50 value from 19.4 μM in vector‐expressing cells to 44.5 μM in IGF2BP2‐overexpressing cells, suggesting the potential contribution of IGF2BP2 in conferring enzalutamide resistance to CRPC cells (Figure 4D).

These findings underscore the role of IGF2BP2 in modulating AR‐V7 expression, influencing downstream target genes, and contributing to enzalutamide resistance in CRPC cells.

### 2.5 KH3 and KH4 Domains of IGF2BP2 are Critical for AR‐V7 Expression

**FIGURE 5:**
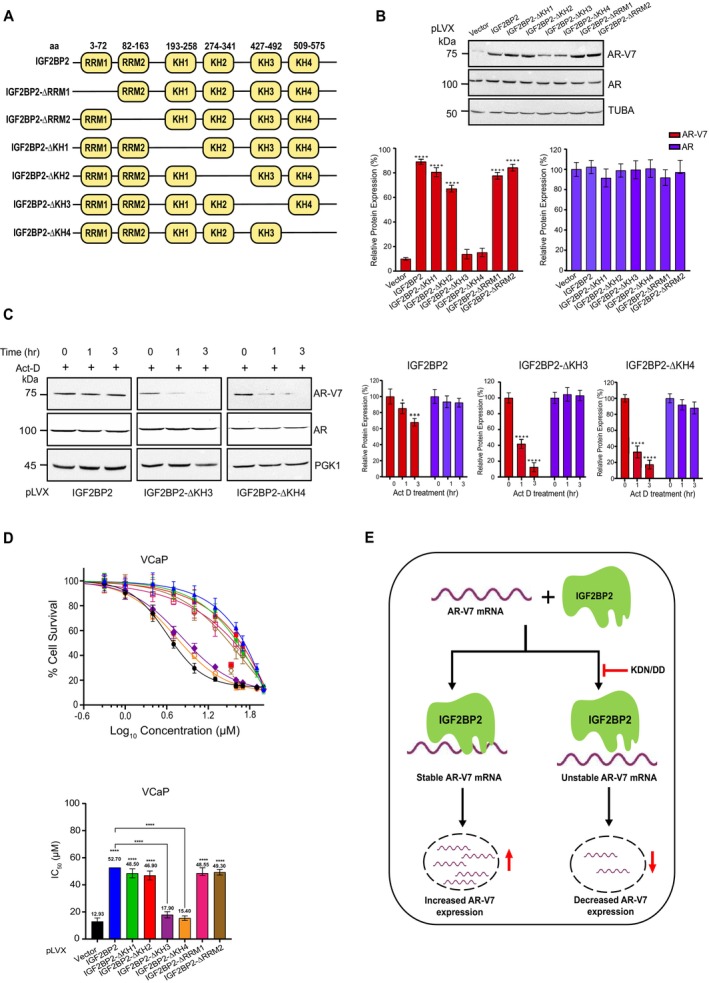
KH3 and KH4 domains of IGF2BP2 are critical for AR‐V7 expression. A. Schematic representation of different domains present in IGF2BP2. B. Immunoblot analysis of AR‐V7 and AR in VCaP cells overexpressing indicated domain‐deleted IGF2BP2 proteins relative to vector‐overexpressing cells. Tubulin was used as loading control. Densitometric analysis for relative AR‐V7 (red bars) and AR (blue bars) expression was performed using Image J software and normalized to TUBA expression. Data are the mean ± SD. *p < 0.05, **p < 0.01, ***p < 0.001. C. Immunoblot analysis of indicated proteins in VCaP cells expressing either IGF2BP2‐KH3 or ‐KH4 domain‐deletion proteins relative to IGF2BP2‐expressing cells following actinomycin‐D (5 μg/mL) treatment for 0, 1, and 3 h. (*n* = 3 biological replicates/group). Densitometric analysis for relative AR‐V7 (red bars) and AR (blue bars) expression was performed using Image J software and normalized to PGK1 expression. Data are the mean ± SD. *p < 0.05, **p < 0.01, ***p < 0.001. D. MTT assay to determine IC50 of enzalutamide in VCaP cells expressing indicated domain‐deleted IGF2BP2 proteins relative to vector‐expressing cells. DMSO was used as control. The data are shown as the mean ± SD; *n* = 3 independent experiments, and two‐tailed student *t*‐test was used to evaluate statistical the significance. *p < 0.05, **p < 0.01, ***p < 0.001, ****p < 0.0001 E. Model summarizing the effect of IGF2BP2 on AR‐V7 mRNA stability.

IGF2BP2 protein comprises of two RNA recognition motifs (RRM) and four K homology (KH) domains that govern its RNA‐binding properties (Figure 5A). To pinpoint the domain(s) that might be crucial for modulating AR‐V7 expression, we systematically constructed IGF2BP2 domain‐deletion mutants (i.e., two RRM domains, and the four KH domains) (Figure 5A). Immunoblot analysis in VCaP cells expressing the various deletion constructs revealed that the cells expressing IGF2BP2 protein lacking KH3 and KH4 domains fail to upregulate AR‐V7 expression relative to the WT, KH1, KH2, RRM1, and RRM2 deletion‐expressing cells (Figure 5B). Importantly, expression of these mutants had no discernible effect on AR expression, pointing toward the specificity of KH3 and KH4 domains in modulating AR‐V7 expression.

Next, we wanted to check if KH3 and KH4 domains are also responsible for maintaining AR‐V7 mRNA stability. We treated VCaP cells expressing various domain deletion constructs with actinomycin‐D for 0, 1, and 3 h and performed immunoblot analysis. The results revealed ~50%–60% decrease in AR‐V7 protein expression 3 h post actinomycin‐D treatment in cells expressing KH3 and KH4 deletion constructs relative to WT, KH1, KH2, RRM1, and RRM2 deletion‐expressing cells (Figures 5C and S7). Consistent with earlier observations, none of the deletions had any impact on AR stability (Figures 5C and S7). These results suggest that IGF2BP2 binds to AR‐V7 mRNA through its KH3 and KH4 domains to regulate its stability and expression.

Next, we investigated if presence of KH3 and KH4 domains in IGF2BP2 plays a role in imparting enzalutamide resistance to CRPC cells. For this, we treated VCaP cells expressing vector, WT‐IGF2BP2, and various domain deletion constructs with varying concentrations of enzalutamide and measured the IC50 using MTT assay. Notably, there is marked increase in IC50 value for enzalutamide in cells expressing IGF2BP2, RRM1, RRM2, KH1, or KH2 deletion constructs while the cells expressing KH3 or KH4 domain‐deletion mutants did not exhibit any significant increase in IC50 values relative to vector‐expressing cells. The IC50 value for enzalutamide in IGF2BP2‐expressing cells is 52.7 μM, however it decreased to 17.9 μM and 15.4 μM in KH3 and KH4 overexpressing cells which are comparable to 12.93 μM in vector‐expressing cells (Figure 5D). Collectively, these results demonstrate that the presence of KH3 and KH4 domains in IGF2BP2 is critical for regulating AR‐V7 stability, expression, and enzalutamide resistance in CRPC cells.

## 3 Discussion

The aggressiveness of prostate cancer, leading to metastatic lesions and CRPC development, underscores the necessity for understanding the signaling pathways and molecules driving progression and drug resistance. Previous studies have highlighted the role of LBD‐truncated AR‐Vs in CRPC [7, 37], with the constitutively active splice variant AR‐V7 being a major driver of tumorigenesis and resistance to anti‐androgens like enzalutamide and abiraterone highlighting the urgency to target AR‐V7 [3, 37, 38].

IGF2BP2 belongs to the family of IGF2 RBPs, which are critical regulators of growth and development in various cancer types. Recent studies have demonstrated its role as an m6A reader, playing a causal role in the development and progression of different cancers including hepatocellular carcinoma, glioblastoma, liposarcoma, pancreatic carcinoma, and ovarian carcinoma [27, 28, 29]. It comprises of six highly conserved RNA‐binding domains (RBDs) that play an important role in the recognition, binding, and stabilization of IGF2BP2‐RNA complexes and also mediate the recruitment of other RBPs [25]. However, the role of IGF2BP2 in CRPC development and progression has not been previously reported. Our TCGA data analysis demonstrated that IGF2BP2 is significantly upregulated in CRPC patient samples suggesting its association with disease progression and poor survival. This upregulation in IGF2BP2 levels is associated with elevated Gleason score suggesting a potential correlation between IGF2BP2 expression and relative aggressiveness of CRPC. Based on the previous studies identifying the role of IGF2BP2 in various cancer types and our analysis of both primary prostate and CRPC patient samples, we postulated that the upregulation of IGF2BP2 might be linked to CRPC progression and enzalutamide resistance.

Here, we have discovered that IGF2BP2 downregulation significantly reduces AR‐V7 expression inside cells without affecting the AR levels, consequently affecting CRPC progression. Moreover, the expression of AR‐V7 target genes is also abrogated upon IGF2BP2 silencing suggesting the broader impact of IGF2BP2 on CRPC progression. Furthermore, we show that IGF2BP2 directly and selectively binds to the ISE region of AR‐V7 mRNA and regulates its stability which is consistent with previous reports showing direct binding of IGF2BP2 to RNA [39, 40]. The enhanced mRNA stability leads to persistent AR‐V7 expression inside cells, thereby contributing to CRPC progression and enzalutamide resistance, in line with previous studies demonstrating the role of IGF2BP2 in regulating cancer progression by stabilizing various mRNA and lncRNA transcripts [39, 40, 41].

Interestingly, our results also demonstrate that IGF2BP2‐mediated regulation of AR‐V7 expression and stability depends upon the presence of intact KH3 and KH4 domains in IGF2BP2. This points toward the ability of these domains to directly bind to AR‐V7 mRNA, which is consistent with the ability of KH domains to confer sequence‐specific RNA recognition ability to IGF2BP2 and reports showing that IGF2BP1 regulates mRNA stability of a large number of targets via its KH3KH4 domains [25, 42]. Interestingly, we observe that the presence of intact KH3 and KH4 domains also positively correlates with enzalutamide resistance, highlighting the importance of sequence‐specific interaction of these domain scaffolds with AR‐V7 mRNA in regulating drug resistance and disease progression. This observation is also consistent with previous studies demonstrating that IGF2BP1 regulates m6A methylation of target transcripts in cancer cells via KH3KH4 di‐domain [25].

In summary, our results, for the first time, demonstrate that IGF2BP2 regulates AR‐V7 expression by directly binding to the AR‐V7 pre‐mRNA. Altering IGF2BP2 expression prevents tumor cell proliferation and resensitizes the cells to ADT. Our mechanistic analysis reveals that KH3 and KH4 domains in IGF2BP2 are responsible for its interaction with AR‐V7 mRNA thereby helping regulate its stability. Taken together, the identification of IGF2BP2 as a key modulator of AR‐V7 expression positions it as a potent therapeutic target for future drug discovery efforts.

### 3.1 Limitations and Future Directions

Our study sheds light on the role of IGF2BP2 in regulating AR‐V7 stability and its implication in enzalutamide resistance. However, additional studies would be required to precisely elucidate the specific sequence(s) within the AR‐V7 ISE region that are bound by IGF2BP2 to regulate its stability. Future experiments involving comprehensive mutagenesis of AR‐V7 mRNA will be required to pinpoint the exact binding sequence(s) of IGF2BP2 and its KH3 and KH4 domains on AR‐V7 mRNA. Elucidating IGF2BP2 crystal structure in complex with AR‐V7 mRNA could further aid these experiments and provide detailed insights into the mechanism of action of IGF2BP2. Moreover, while our cell line studies demonstrate that IGF2BP2 inhibition can mitigate oncogenic AR‐V7 signaling and enzalutamide resistance, it would be worth corroborating these findings in vivo models of CRPC.

## 4 Materials and Methods

### 4.1 Differential Gene Expression and Gleason Analysis

Raw count data of WCDT‐MCRPC and TCGA‐PRAD datasets comprising CRPC (*n* = 99), primary prostate cancer samples (*n* = 498), and normal prostate samples (*n* = 52) were downloaded from TCGA genomic data commons data portal (https://portal.gdc.cancer.gov/). Differential gene expression analysis of CRPC vs. primary prostate and CRPC vs. healthy controls was performed using DESeq2 package in R programming studio. DESeq2 employs an internal normalization method known as: median of ratios method, which calculates size factors for the normalization of raw counts, ensuring precise comparison of gene expression across diverse samples, even if they are sequenced at varying depths [1] . We used absolute log2 fold change > 0.5 and adjusted p‐value < 0.05 as cut‐off criteria to shortlist differentially expressed genes.

For Gleason analysis, metastatic prostate adenocarcinoma (PRAD_SU2C_2019) datasets containing FPKM transcript data information along with corresponding Gleason scores for 168 CRPC patients were downloaded from cBioPortal (https://www.cbioportal.org/). The data was plotted and statistical analysis was performed using GraphPad PRISM software (https://www.graphpad.com).

### 4.2 Cell Culture

HEK293T, VCaP, and 22Rv1 cells were purchased from ATCC. LnCaP95 cells were a kind gift from Dr. Jun Luo, Johns Hopkins School of Medicine, Baltimore, MD. HEK293T and VCaP cells were cultured in DMEM media (HiMedia), and 22Rv1 cells were cultured in RPMI1640 media (HiMedia) supplemented with Pen‐strep antibiotics, L‐glutamine, and 10% FBS. LnCaP95 cells were cultured in Phenol‐red free RPMI1640 media (Himedia) supplemented with Pen‐strep antibiotics, L‐glutamine, and 10% charcoal‐stripped FBS (CFBS). All the cell lines were maintained in presence of 5% CO2 at 37°C and were tested for any bacteria, fungi, and mycoplasma contamination.

### 4.3 Viral Production and Generation of Stable Knockdown Cells

For lentivirus production, 2 X 10^6^ HEK293T cells were seeded in individual wells of a 6‐well plate and incubated for 24 h at 37°C after which they were co‐transfected with lentiviral packaging plasmids along with IGF2BP2 shRNA plasmid using Effectene as a transfection reagent. 48 h post‐transfection, the viral supernatant was filtered using a 0.45 μM filter. For stable knockdown of IGF2BP2, 2 X 10^6^ cells were infected either with non‐silencing shRNA or IGF2BP2 shRNA. Forty‐eight hours post‐infection, cells were selected using appropriate puromycin concentrations (0.5 μg/mL for 22Rv1 and 2 μg/mL for VCaP and LnCaP95) for 4–5 days. Cells were cultured for another 2–3 days after puromycin removal before being processed for further experiments. IGF2BP2 shRNA sequences used in this study are listed in Supplementary Table 1.

### 4.4 RNA Preparation, cDNA Preparation and Quantitative PCR (qPCR) Analysis

Total RNA was isolated from 1.5 X 10^6^ cells using TRIzol (Invitrogen, USA) and cDNA synthesis was performed using the Superscript II cDNA Synthesis Kit (Invitrogen, USA). qRT‐PCR was performed using the PowerUp SYBR Green Master Mix, with gene‐specific primers as per manufacturer's instructions. Expression of target mRNAs was quantified using ΔΔCT method relative to the house‐keeping gene ACTB. Primer sequences for all the genes analyzed in this study are listed in Supplementary Table 2.

### 4.5 Protein Extraction and Immunoblot Analysis

For total protein cell lysate preparation, 2.0 X 10^6^ cells were harvested using cell scraper and lysed for 10 min using RIPA buffer (20 mM Tris–HCl (Affymetrix), pH ‐7.5, 150 mM NaCl (Affymetrix), 1 mM EDTA (Affymetrix), 1 mM EGTA (HiMedia), 1% NP‐40, 1% Sodium deoxycholate (Sigma), 1% sodium orthovanadate (sigma) containing protease (Roche) and phosphatase inhibitor cocktail), following which it was centrifuged at 14,000 rpm for 30 min. Protein concentration in the clarified supernatant using Bradford Protein Assay Reagent (BioRad Laboratories). 40 μg of protein lysate was loaded onto SDS‐PAGE gel using a Mini‐Protean System (BioRad) and transferred onto nitrocellulose membrane (BioRad 0.2 μm) for 3 h at 250 mA. The membranes were blocked in Tris‐buffered saline containing 0.1% Tween 20 (TBST) containing 5% skimmed milk for 1 h following which primary antibodies (AR‐V7: AG10008 (Precision antibody); AR: 06–680 (Merck Millipore); PGK1: ab38007 (abcam) Tubulin: ab15246 (abcam) were added overnight at 4°C. After probing with primary antibody, membranes were washed using TBST and then incubated with HRP‐conjugated secondary antibody at room temperature for 1 h. Blots were finally developed using chemiluminescent substrate (SuperSignal West Pico PLUS Chemiluminescent Substrate, Thermo Fisher Scientific) in chemiDoc imaging system (BioRad Laboratories).

### 4.6 Plasmid Construction

Human IGF2BP2 was cloned into p3XFLAG‐myc‐CMV 26 (Sigma) and pLVX‐E1Fα‐IRES‐mCherry‐Puro (Takara Bio) to perform the experiments associated with its ectopic expression. RRM domain truncations (IGF2BP2‐ΔRRM) and KH domain truncations (IGF2BP2‐ΔKH) were generated by performing overlap PCR reaction [1] using primers listed in supplementary table 3 and cloned in p3XFLAG‐myc‐CMV 26 and pLVX‐E1Fα‐IRES‐mCherry‐Puro between EcoRI and XbaI sites. All the PCR products and clones were verified by DNA sequencing.

### 4.7 mRNA Stability Assay

1.5 X 10^6^ cells infected with either NS, IGF2BP2 shRNA, or various IGF2BP2‐expression plasmids were seeded in individual wells of a 6‐well plate and incubated till 80% confluency was achieved. Subsequently, the cells were treated with 5 μg/mL actinomycin‐D (sigma) and harvested at 0 h, 1 h, and 3 h timepoints. The whole‐cell protein lysate was prepared and analyzed using western blotting. The protein degradation was quantified by calculating the band intensity using densitometry analysis. All the data was derived from three independent biological replicates.

### 4.8 RNA Immunoprecipitation and qRT‐PCR Analysis

5X10^6^ IGF2BP2 overexpressing VCaP cells were washed with ice‐cold PBS post‐infection followed by lysis of cells using immunoprecipitation (IP) lysis buffer containing 50 mM Tris‐Cl (pH 7.4), 250 mM NaCl, 5 mM EDTA, 0.5 mM DTT, 0.5% Triton X‐100, 1X protease inhibitor (Roche)) on ice, following which the lysate was subsequently centrifuged at 14,000 x g for 30 min at 4°C. The clarified supernatant was carefully removed and 1 mg/sample lysate was incubated with 1ug of indicated antibodies at 4°C. IgG antibody was used as negative control. 20 μL Protein G‐coated Dynabeads (Life Technologies) were added to the mixture, incubated at 4°C for 2 h, separated using magnetic stand, and washed 3X times with IP lysis buffer. Bound RNA was eluted from the dynabeads using TRIzol, reverse transcribed and the enrichment of IGF2BP2 on ESE and ISE regions was estimated using qRT‐PCR. The primers used for the analysis are listed Supplementary Table 2.

### 4.9 Wound‐Healing Assay

1.5 X 10^6^ VCaP cells were infected with the indicated shRNAs in a 6‐well plate and incubated until 80% confluency was achieved. After the cells formed a confluent monolayer, scratches were performed using 200 μL tip and detached cells were removed by giving PBS wash following which fresh culture media was added. The closure of scratch was analyzed under the microscope until the scratch was completely covered in the control well. Images were taken under the microscope (Nikon DS‐F*i*3 microscope). Difference in wound width was measured to calculate the cell migration ability. All the data was derived from three independent biological replicates.

### 4.10 Colony‐Formation Assay

5 X 10^3^, VCaP, LnCaP95, or 22Rv1 cells infected with various viruses were seeded in individual wells of a 6‐well plate and incubated at 37°C until distinct colonies were formed. Cells were washed with PBS and stained with 0.01% crystal violet. Positively stained colonies were observed under the microscope and imaged.

### 4.11 Soft Agar Assay

The effect of IGF2BP2 knockdown on anchorage‐independent growth of 22Rv1 and LnCaP95 cells was monitored in a 6‐well plate coated with basal layer of 1.2% Nobel agar and RPMI‐1640 containing 20% FBS. Stable knockdown cells were seeded at 5000 cells/well density in media mixed with 0.6% Nobel agar. After the agar was set, 1 mL RPMI 1640 + 10% FBS media was added to each well. 3 weeks later, the macroscopic colonies formed on the plate were stained with 0.001% crystal violet and imaged using a phase contrast microscope with a 10X objective.

### 4.12 Cell Proliferation Assay

5 X 10^3^, 22Rv1, or LnCaP95 cells (NS and IGF2BP2 knockdown) were seeded in each well of a 96‐well plate with a density. Following 24 h incubation, cells were treated with varying concentrations of enzalutamide (0.5 μM—100 μM) using DMSO as a control. Cell proliferation post‐enzalutamide treatment in IGF2BP2 knockdown cells was analyzed using MTT reagent, and the absorbance value was measured at OD 595 nm using a microplate reader (BioRad). Each group was analyzed in triplicate.

### 4.13 Statistical Analysis

Statistical analysis was performed using t‐test (two‐tailed unpaired) and ANOVA in GraphPad Prism (www.graphpad.com). All the experiments included at least three biological replicates unless stated otherwise. Data is represented as mean ± S.D. and the statistical significance is denoted by *p*‐values.

## Author Contributions

**Taruna Saini:** conceptualization (lead), data curation (lead), formal analysis (lead), investigation (lead), methodology (lead), visualization (lead), writing – original draft (lead), writing – review and editing (lead). **Devesh Srivastava:** data curation (supporting), formal analysis (supporting), visualization (supporting), writing – original draft (supporting), writing – review and editing (supporting). **Rajnikant Raut:** conceptualization (supporting), data curation (supporting), methodology (supporting), visualization (supporting), writing – original draft (supporting), writing – review and editing (supporting). **Parul Mishra:** conceptualization (equal), funding acquisition (equal), project administration (equal), resources (equal), supervision (supporting), writing – original draft (equal), writing – review and editing (equal). **Ashish Misra:** conceptualization (equal), funding acquisition (lead), project administration (lead), resources (lead), supervision (lead), writing – original draft (equal), writing – review and editing (equal).

## Consent

The authors have nothing to report.

## Conflicts of Interest

The authors declare no conflicts of interest.

## Supporting information


**Data S1** supporting Information

## Data Availability

All data generated during this study are included in this published article and its supplementary files.
