# Supplementary material for: Insulin‐Like Growth Factor 2 mRNA‐Binding Protein 2 (IGF2BP2) Promotes Castration‐Resistant Prostate Cancer Progression by Regulating AR‐V7 mRNA Stability
Source: Cancer Rep (Hoboken). 2025 Feb 13;8(2):e70096. doi: 10.1002/cnr2.70096 (PMC11825379; doi:10.1002/cnr2.70096)
Supplement: Supplementary file 1 — Data S1 supporting Information [file CNR2-8-e70096-s001.docx]

**Supplementary Figures**

**Figure S1**

**
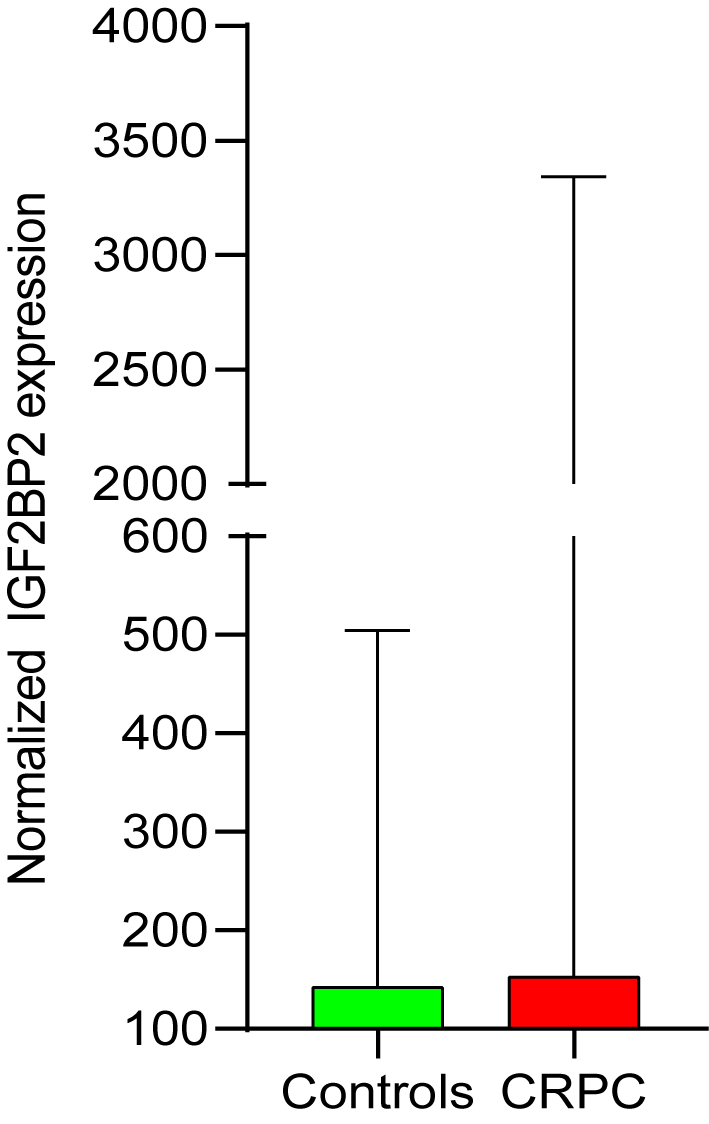
**

Figure S1. Comparative expression of IGF2BP2 in normal patients (n= 52) versus metastatic CRPC patients (n=99) from TCGA-PRAD and WCDT-MCRPC datasets (*p*=0.013 and logFC=0.765). Gene expression is graphically represented as box and whiskers plot with outliers.

**Figure S2**

**
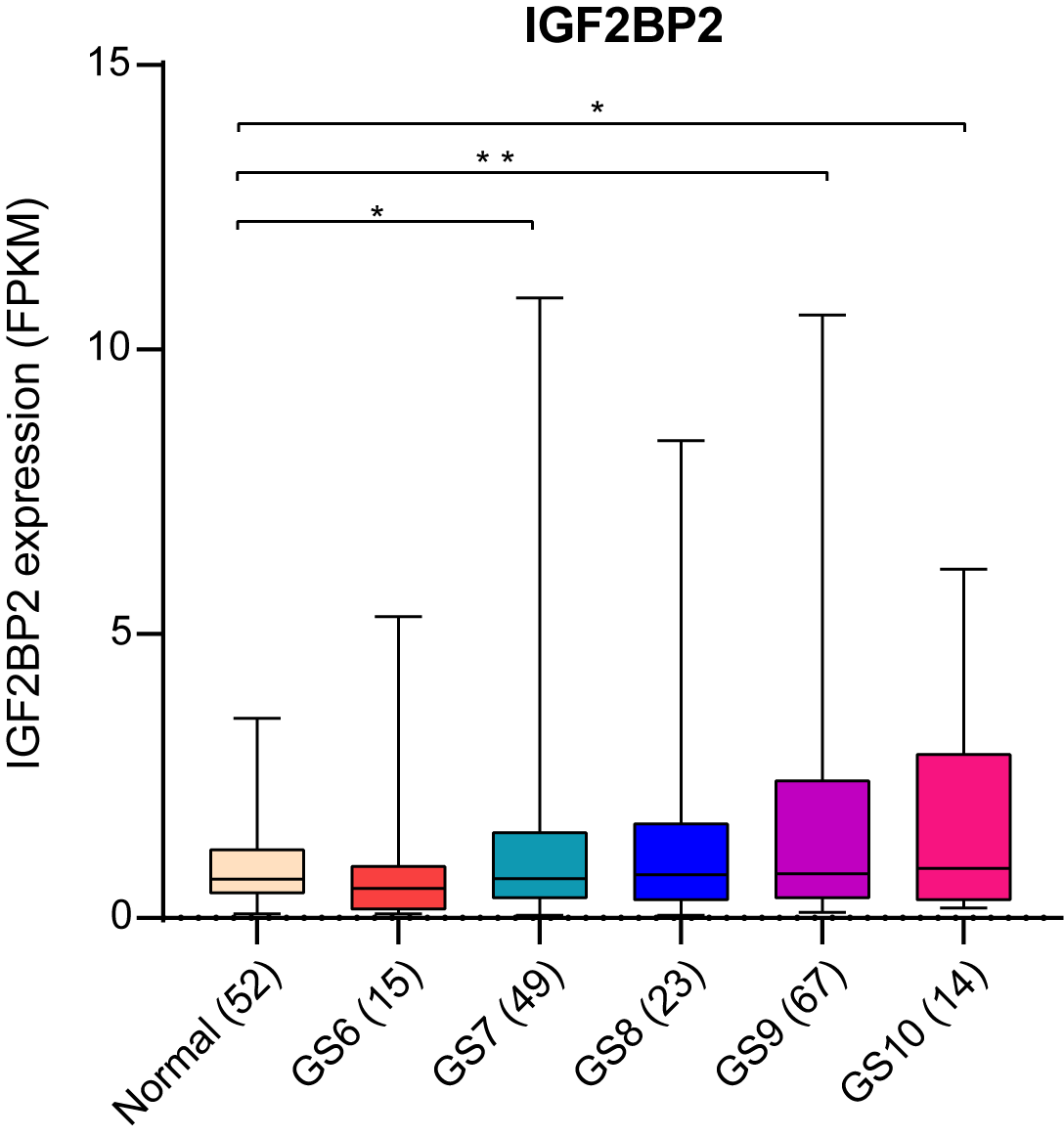
**

Figure S2. Box plots showing IGF2BP2 mRNA level in clinical samples of normal versus Gleason 6, 7, 8 9, 10 score categories obtained from prad_su2c_2019 study. (p-value: *<0.05, **<0.01)


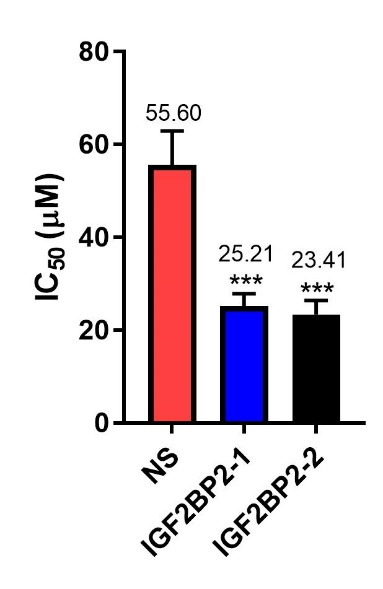
**Figure S3**

**Figure S3:** MTT assay to determine IC50 of bicalutamide in 22Rv1 cell lines expressing NS, IGF2BP2-1 and IGF2BP2-1 shRNAs. The data are shown as the mean ± SD; *n* = 3 independent experiments, two-tailed student’s t-test, (*** p<0.001).

**Figure S4**


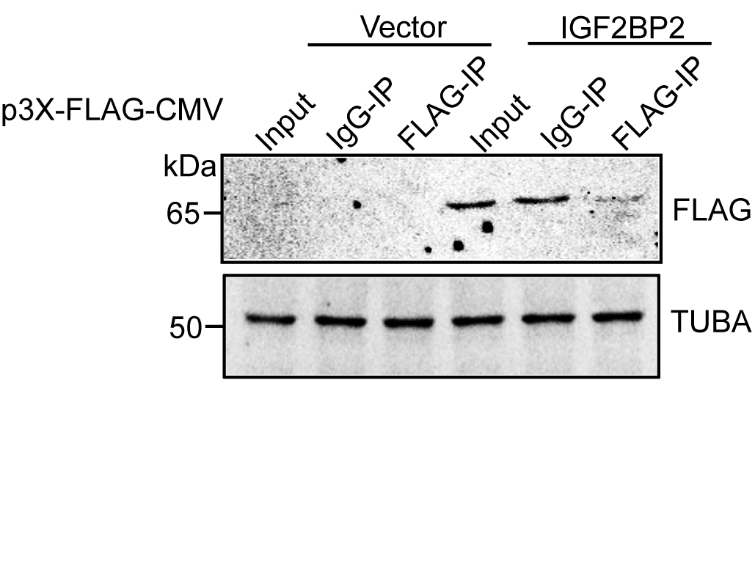


Figure S4. Western blot analysis in the flow-through after anti-FLAG immunoprecipitation in VCaP cells were transiently transfected with either FLAG-IGF2BP2 or empty vector for 72 hours. All immunoprecipitation experiments were performed in biological triplicates.

**Figure S5**


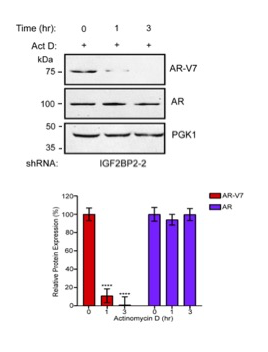


Figure S5. Expression of AR-V7, AR and PGK1 was determined by immunoblot analysis after Actinomycin D treatment (5 μg/mL) at indicated time in IGF2BP2 knockdown 22Rv1 cells. Densitometric analysis of relative expression of AR-V7 and AR was performed using Image J software and normalised by the expression of control. Data are the mean± SEM. **p* < 0.05, *** p < 0.001

**Figure S6**


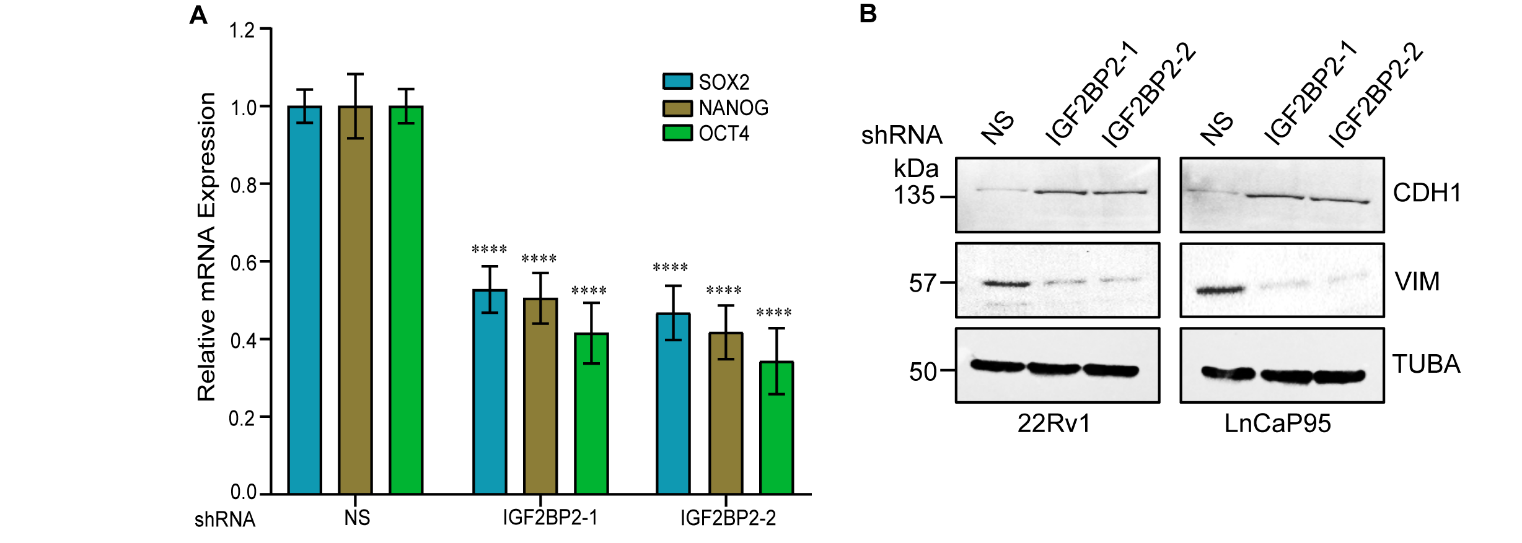


Figure S6. (A) Quantitative gene expression analysis of stem cell genes Sox2, Nanog and Oct-4 by performing qRT-PCR in IGF2BP2 depleted 22Rv1 cells; (B) Protein expression analysis of common EMT markers E-cadherin and Vimentin in IGF2BP2 knockdown 22Rv1 and LnCaP95 cells. Tubulin was used as a loading control.

**Figure S7**


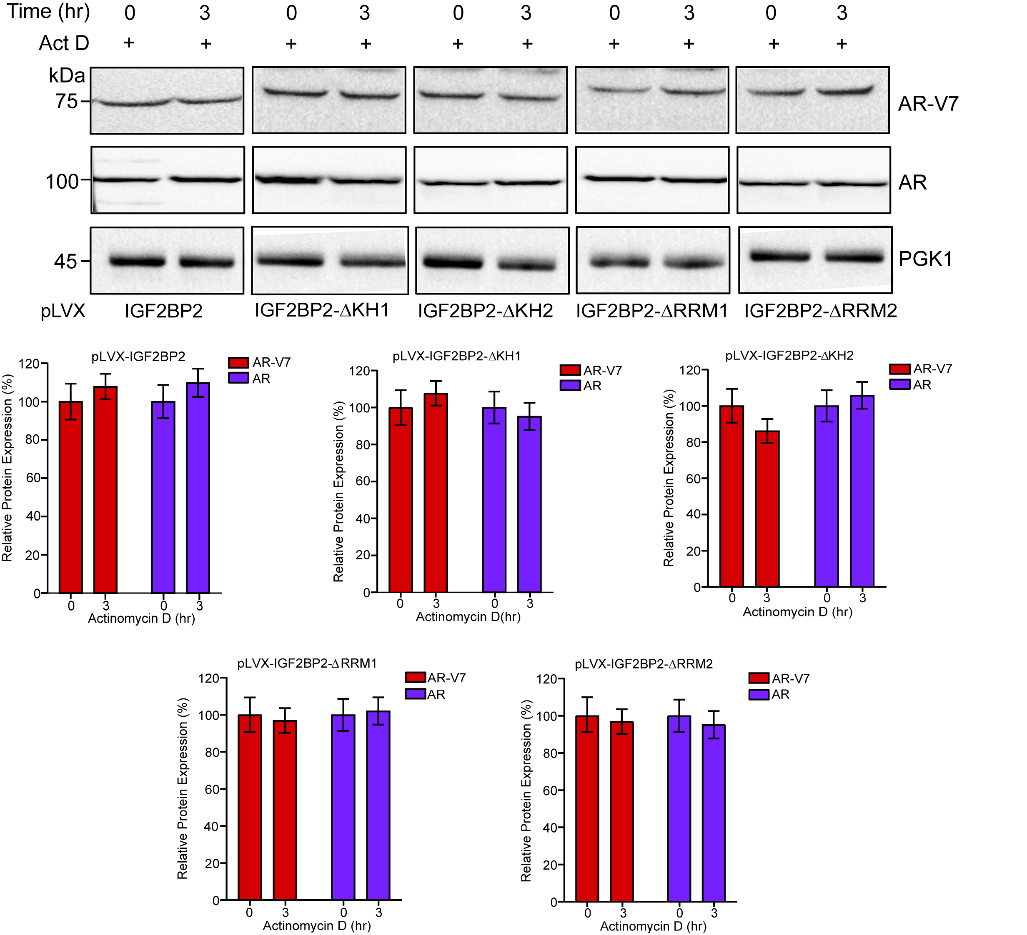


Figure S7. Consistent AR-V7 and AR protein expression on overexpressing full-length IGF2BP2 and KH1-2 and RRM1-2 truncated IGF2BP2 constructs in VCaP cells. Densitometric analysis of relative expression of AR-V7 and AR was performed using Image J software and normalised by the expression of control. Each value in the data represents mean± SEM.

**Supplementary Tables**

**Table S1. List of IGF2BP2 shRNA**

| **SEQUENCE** | **TARGET REGION** |
| --- | --- |
| CGGATCTTTGGGAAACTGAAA | KH3 DOMAIN |
| CTTAACCAGTGCAGAAGTCAT | KH4 DOMAIN |

**Table S2. List of RT-qPCR primers**

| **GENE NAME** | **SEQUENCE** |
| --- | --- |
| AR | F: 5′ TCTTGTCGTCTTCGGAAATGT 3′  R: 5′ AAGCCTCTCCTTCCTCCTGTA 3′ |
| AR-V7 | F: 5′ CGGAAATGTTATGAAGCAGGGATGA 3′  R: 5′ CTGGTCATTTTGAGATGCTTGCAAT 3′ |
| IGF2BP2 | F: 5′ AGTGGAATTGCATGGGAAAATCA 3′  R: 5′ CAACGGCGGTTTCTGTGTC 3′ |
| KLK3 | F: 5′ AGTGCGAGAAGCATTCCCAAC 3′  R: 5′ CCAGCAAGATCACGCTTTTGTT 3′ |
| TMPRSS2 | F: 5′ ATGAAAACCATGGATACCAACCG 3′  R: 5′ GAGAATCCATCTTGAGGTGC 3′ |
| UBE2C | F: 5′ TGCCCTGTATAGATGTCAGGA 3′  R: 5′ GGGACTATCAATGTTGGGTTCT 3′ |
| CDC25A | F: 5′ CAAACCTTGACAACCGATG 3′  R: 5′ ACACTGACCAGTCGTGGAG 3′ |
| UGT2B17 | F: 5′ ACCCAGCCAAACCCTTGCCTAA 3′  R: 5′ GGCTGATGCAATCATGTTGGCAC 3′ |
| ELK1 | F: 5′ CAGCCAGAGGTGTCTGTTACC 3′  R: 5′ GAGCGCATGTACTCGTTCC 3′ |
| CDK1 | F: 5′ CCTAGCATCCCATGTCAAAAACTTGG 3′  R: 5′ TGATTCAGTGCCATTTTGCCAGA 3′ |
| CCN2A | F: 5′ GAAGACGAGACGGGTTGCA 3′  R: 5′ AGGAGGAACGGTGACATGCT 3′ |
| SOX2 | F: 5′ GAGCTTTGCAGGAAGTTTGC 3′  R: 5′ GCAAGAAGCCTCTCCTTGAA 3′ |
| NANOG | F: 5′ ACCTTGGCTGCCGTCTCTGG 3′  R: 5′ AGCAAAGCCTCCCAATCCCAAACA 3′ |
| OCT-4 | F: 5′ TTTTGGTACCCCAGGCTATG 3′  R: 5′ GCAGGCACCTCAGTTTGAAT 3′ |

**Table S3. List of Cloning primers**

| **GENE NAME** | **SEQUENCE** |
| --- | --- |
| IGF2BP2 | F: 5′ CAAAAGCTTATGGCGACGGAGCATCCC 3′  R: 5′ CAAGCGGCCGCAAACTTGAACTCCTTATATTTCTTG 3′ |
| IGF2BP2-ΔRRM1 | F: 5′ CGGGAATTCATGAAAAAGCTAAGGAGCAGG 3′  R: 5′ CAAGCGGCCGCAAACTTGAACTCCTTATATTTCTTG 3′ |
| IGF2BP2- ΔRRM2 | F: 5′ AAAAAGCTAAGGAGCGAAGAGGTGAGCTCCCC 3′  R: 5′ GGGGAGCTCACCTCTTCGCTCCTTAGCTTTTT 3′ |
| IGF2BP2- ΔKH1 | F: 5′ AGGCCAGACAGATTCTTGAAATCATGCAGAA 3′  R: 5′ TTCTGCATGATTTCAAGAATCGTCTGGCCCT 3′ |
| IGF2BP2- ΔKH2 | F: 5′ GACCAAACTAGCCGAAATGAAGAAGCTGC 3′  R: 5′ GCAGCTTCTTCATTTCGGCTAGTTTGGTC 3′ |
| IGF2BP2- ΔKH3 | F: 5′ CACTCTTATCCAGAGTTTGGGAAACTGAAAGAGG 3′  R: 5′ CCTCTTTCAGTTTCCCAAACTCTGGATAAGAGTG 3′ |
| IGF2BP2- ΔKH4 | F: 5′ CCCCAAAGAAGAAGTGGAAATTGTACAACAGGTG 3′  R: 5′ CACCTGTTGTACAATTTCCACTTCTTCTTTGGGG 3′ |
